# Myth-busting the provider-user relationship for digital sequence information

**DOI:** 10.1093/gigascience/giab085

**Published:** 2021-12-29

**Authors:** Amber Hartman Scholz, Matthias Lange, Pia Habekost, Paul Oldham, Ibon Cancio, Guy Cochrane, Jens Freitag

**Affiliations:** Leibniz Institute DSMZ-German Collection of Microorganisms and Cell Cultures GmbH, Department of Microbial Ecology and Diversity, 38124 Braunschweig, Germany; Leibniz Institute of Plant Genetics and Crop Plant Research, Department of Breeding Research, OT Gatersleben, 06466 Seeland, Germany; Leibniz Institute of Plant Genetics and Crop Plant Research, Department of Breeding Research, OT Gatersleben, 06466 Seeland, Germany; Manchester Institute of Innovation Research, Alliance Manchester Business School, Manchester University, Manchester, M15 6PB, UK; Research Centre for Experimental Marine Biology and Biotechnology of Plentzia (PiE-UPV/EHU), University of the Basque Country (UPV/EHU), EMBRC-Spain, E-48620, Plentzia, Spain; European Molecular Biology Laboratory, European Bioinformatics Institute (EMBL-EBI), Wellcome Genome Campus, Hinxton, Cambridge, CB10 1SD, UK; Leibniz Institute of Plant Genetics and Crop Plant Research, Department of Breeding Research, OT Gatersleben, 06466 Seeland, Germany

**Keywords:** UN Convention on Biological Diversity, access and benefit sharing, digital sequence information, provider user, Nagoya Protocol, INSDC

## Abstract

**Background:**

The United Nations Convention on Biological Diversity (CBD) formally recognized the sovereign rights of nations over their biological diversity. Implicit within the treaty is the idea that mega-biodiverse countries will provide genetic resources and grant access to them and scientists in high-income countries will use these resources and share back benefits. However, little research has been conducted on how this framework is reflected in real-life scientific practice.

**Result:**

Currently, parties to the CBD are debating whether digital sequence information (DSI) should be regulated under a new benefit-sharing framework. At this critical time point in the upcoming international negotiations, we test the fundamental hypothesis of provision and use of DSI by looking at the global patterns of access and use in scientific publications.

**Conclusion:**

Our data reject the provider-user relationship and suggest a far more complex information flow for DSI. Therefore, any new policy decisions on DSI should be aware of the high level of use of DSI across low- and middle-income countries and seek to preserve open access to this crucial common good.

## Background

The Convention on Biological Diversity (CBD) is the international policy mechanism to reduce species, habitat, and ecosystem loss on this planet. The three overarching goals of the CBD, agreed upon in 1992, are conservation of biodiversity, sustainable use of this biodiversity, and fair and equitable benefit sharing from genetic resources. The third goal represents a political “balancing act” with the first two goals because it is intended to incentivize access and use of genetic resources (GR) so that benefits from use of biodiversity will flow back to the providing country, thus encouraging conservation, and supporting the first two goals.

Although it is officially recognized by parties to the CBD that all countries are both users and providers of GR, in practice, most low- and middle-income countries (LMICs) see themselves predominantly as providers and, conversely, many high-income countries (HICs) view themselves as users [1]. While the CBD originally envisioned a facilitation mechanism for access to GR, the Nagoya Protocol (negotiated in 2010) codified a bilateral system in which a single country gives permission to a single user, which has perpetuated the provider-user paradigm [2]. In fact, the complex legal landscape that has resulted from the post-2010 implementation of the Nagoya Protocol reflects this. HICs often focus on user compliance [3] and LMICs focus on access laws even though every country should theoretically be responsible for user checks [4]. (Countries are not bound by the Nagoya Protocol to regulate access.) For example, to our knowledge, to date only high-income countries have implemented user compliance mechanisms (i.e., laws that check whether users have complied) with provider country laws, most notably the European Union [5] and Japan [6].

However, whether patterns of scientific use of GR actually follow these user-provider assumptions is not a question that has received much attention [7]. GR provision and use is difficult to follow because GR sampling and exchanges are not centrally administered or recorded. However, the use and citation of sequence data from GR in scientific publications enables a “proxy” view on provider-user relationships and happens to be itself highly relevant to the current CBD discussions.

In CBD policy circles, nucleotide sequence data (as well as potentially other data types) are known as “digital sequence information” (DSI) [8]. Because of the exponential growth and widespread use and reliance on DSI in the biological sciences, the political question of the hour is whether and how benefit sharing from DSI should be required. This decision is contested but widely expected to be resolved in some form at Part 2 of the 15^th^ Conference of the Parties (COP15). COP15, which has been delayed by the pandemic but tentatively scheduled for late June 2022, will answer whether DSI should be treated like GR, whether monetary and/or non-monetary benefit sharing will be required and documented, and, if so, whether the policy framework for benefit sharing will be bilateral or multilateral [9]. Thus, COP15 will be an important milestone for policymakers and scientists alike, making the question of patterns of use of DSI, presented here, a timely one.

Because many negotiators at the COP15 will be familiar with the bilateral mechanisms of the CBD and its Nagoya Protocol, it is likely that the default preconception around DSI for most negotiators will be the “provider-user dichotomy” assuming a primarily uni-directional (roughly global south to north) provision and use relationship. This is actually a hypothesis that can be tested with data from open-access public DSI databases, in which the country of origin for the DSI can be found, and via publication databases, where use of DSI can be assessed by proxy through the affiliations of the authors, which can be parsed into geographical locations. While keeping in mind the potential shortcomings and accuracy issues [10], here we test this hypothesis and display the results in a free and open data analysis platform with the aim of analyzing whether a real directionality exists from provider country to DSI user country, with LMICs on one side and HICs on the other. The data and their implications are intended to support evidence-based policymaking.

## Data Description

This article is a companion paper to a Data Note published in the same issue of this journal [11], which was also made available as a preprint in bioRxiv. A web application is provided to explore and visualize the dataset [12], and, for clarity, we have compiled here a brief excerpt of the dataset described in the Data Note.

Most scientific journals and funding agencies require that DSI be made freely available, at the latest, by the time of publication. Submissions of sequence data are required by journals as a condition of publication and rely on the use of unique identifier(s) (called accession numbers [ANs]) generated by a member of the International Nucleotide Sequence Database Collaboration (INSDC). During the sequence submission process, metadata associated with the DSI are also submitted including, where appropriate, the country field (data field "/country") which is defined as “locality of isolation of the sequenced sample indicated in terms of political names for nations, oceans or seas, followed by regions and localities.”

At the time of these analyses, there were 17,816,729 sequences in the INSDC with a country tag. Generally, sequences with country information come from a natural environment. For this study, we did not perform subsequent analyses on the taxonomic distribution of these sequences, which was previously assessed in [13]. Access to GR is needed to produce DSI. In this article we use the term “provider” to designate the /country information found in the INSDC and indicate the geographical location from where the GR, and thus indirectly the DSI, originated. (Note that “provider” does not reflect where the sequencing was done or the entity that made the research/funding investment).

For each of the >17.8 million ANs, if a publication was listed in the sequence entry page (within the INSDC database), this was added to the dataset as a “primary” publication. In a parallel step, the European PubMedCentral (ePMC) open-access publication database was text-mined for all >17.8 million sequence ANs. If a publication listed any of these sequences, it was added to the dataset as a “secondary publication.” A total of 117,483 primary and/or secondary publications were included in this analysis. Publications citing the use of DSI are representative of DSI scientific “use.” Double counts were removed. The associated author metadata from the primary and secondary publications was machine read and parsed. The geographical location of the first author was identified where data quality was sufficient. We note that first author information presents a restricted view of author networks that reflects limitations in the availability of full author information. As more author data become available, we anticipate that it will be possible to engage in analysis of author networks. This dataset forms the basis of the DSI “user” geographical locations. Additional quality control, data parsing, table merging, and data visualization steps were required that are further explained in the companion Data Note [11]. We make no further classification under the term “use” because our methods at this stage cannot distinguish among the different types of use of DSI, e.g., commercial versus non-commercial. On average, we expect that many peer-reviewed publications are more likely to derive from non-commercial research.

## Analyses

**Figure 1.**
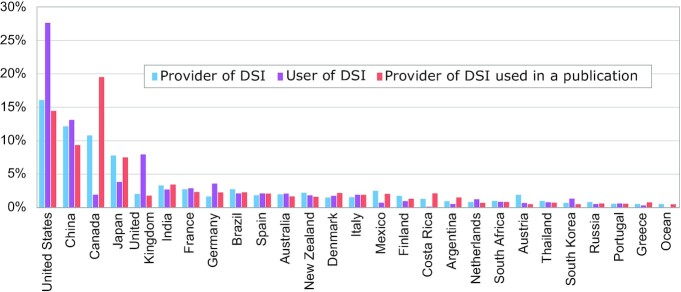
Bar graph comparison of each country's provision of DSI (i.e., where it is the country of origin) for DSI relative to its proportion of users (authors in scientific publications that reference DSI) and the proportion of its DSI cited. An interactive chart is available on the web platform [12] under Graph 1.6.

The first question addressed is which countries are currently providing DSI to and using DSI from the global dataset available through the INSDC (Fig. 1). The largest providers of DSI are currently not LMICs but are the United States, China, Canada, and Japan, providing roughly half of the global dataset. Large middle-income countries such as India and Brazil are in the next wave of providers (Fig. 1, blue bars). In the 2 years since Rohden et al. [13] described this trend for a CBD-commissioned study, the pattern has not significantly changed. However, it is important to note that only 14.6% of all sequences in this INSDC release have country information available. This is down slightly from the 16% observed in the April 2019 global sequence dataset analyzed by Rohden et al. However, this does not necessarily represent a statistical trend. There are multiple factors that could cause this seeming decrease. For example, large deposits of sequences that are not appropriate for country labelling, e.g., human data (which would only rarely have country information associated with them) could grow the dataset and thus decrease the proportion of country-labelled DSI. This question (cause of the 16% vs 15%) was not further investigated.

Once these DSI are made available by provider countries, the natural question is to ask who is using the DSI, i.e., scientists sitting in which countries are publishing (which we call “using”) and citing DSI in a publication (Fig. 1, purple bars). To begin to understand the provider-user relationship, it is also important to understand where the DSI that was *actually* used in a publication comes from, i.e., which countries provided access to the GR (Fig. 1, red bars). To summarize, most DSI is being provided and used by HICs; and DSI use and provision often occur in roughly similar proportions. However, some outliers such as Costa Rica and Argentina do show larger differences between providing and using DSI. These data do not, however, support the idea of a unidirectional provider-user dichotomy.

**Figure 2.**
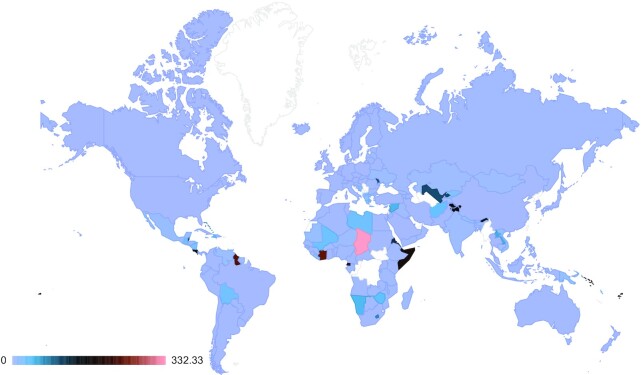
The “self:world” use ratio, which is the relationship between the foreign use (by non-domestic scientists) of a country's DSI (numerator) and the use of its DSI by domestic scientists (denominator). Light blue indicates a balanced use of DSI between foreign and domestic scientists. Pink indicates strong foreign use of DSI with less domestic use. Dark colours (black and red) indicate intermediate values. An interactive chart is available on the web platform under Graph 3.4 [12].

To further understand the real-world provider-user relationship, another angle to examine is the relationship between a country's use of its own “national DSI” (where it was the provider country of the GR) versus its use of DSI from other countries. We call this the “self: world” use ratio where self-use of DSI is divided by use of DSI from all other countries (Fig. 2).

Although there are significant differences in the number of scientists and the volume of use and reuse between LMICs and HICs, the relationship between a country's use of its own DSI and use of the global DSI dataset (all non-national DSI) is relatively homogenous. A few countries have more “foreign use,” i.e., their national scientists use less relative to non-national scientists, such as Chad and Guyana. However, the vast swaths of blue in the map suggest that the ratio between national and foreign use of a country's DSI is relatively even.

At international meetings, including the CBD Conference of the Parties, countries can form negotiating blocs that enable coordination between similar perspectives and sharing of preparation work when developing negotiating positions. These blocs often represent underlying economic similarities between countries. To understand broad trends through a similar lens used in these political discussions, we grouped countries into 3 overarching groups: low-income countries in a group called G-77, middle-income countries known as BRICS (Brazil, Russia, India, China, South Africa), and high-income countries under OECD (Organization of Economic Cooperation and Development). We note that South Africa, Brazil, and India are officially members of the G-77 but for this study, we have only counted them in the BRICS group. These broad groupings, although imperfect, allow for visual representations that could proxy common trends within UN political discussions.

**Figure 3.**
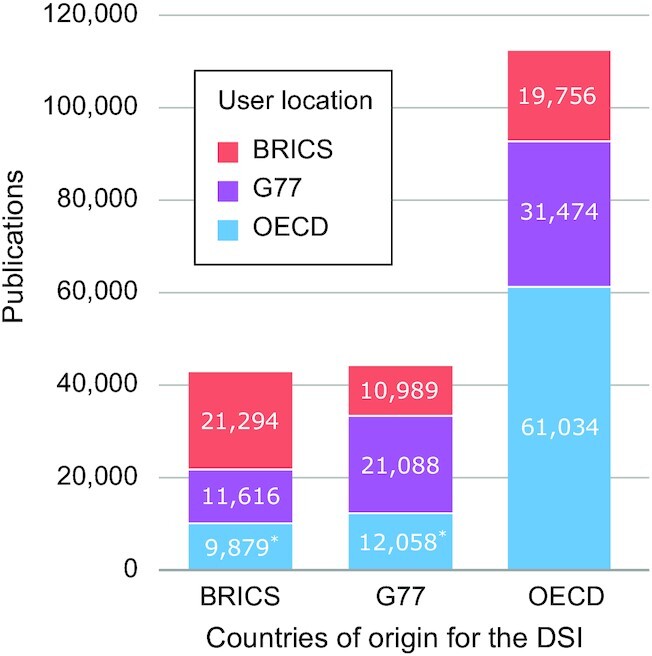
DSI use by economic blocs. On the x-axis is the country of origin of the DSI (i.e., the “/country” field in the sequence database). On the y-axis are the total number of DSI-using publications, where the colour blocks represent the geographical location of the users (authors) grouped by economic blocs (i.e., OECD [proxy for developed countries], G77 [proxy for low-income countries], and BRICS [Brazil, Russia, China, India, and South Africa, proxy for large middle-income countries]). The asterisk indicates the subset of the DSI-based publications where the international call for benefit sharing is most focused and where concerns are heightened. An interactive chart is available on the web platform under Graph 5.1 [12].

Three significant trends can be observed in Fig. 3. First, the largest group of users (counted by a publication not an individual) in each bar always matches the economic bloc of the country of origin of the DSI. For example, the biggest group of users using G77-sourced DSI is G77-located users. This suggests that users tend to use DSI from their own country/bloc rather than from outside. They research and publish more with “locally sourced” DSI. These data again reject the unidirectional provider-use hypothesis. If that hypothesis were true, then OECD users (from HICs) should be the biggest user group in all 3 bars. Instead, OECD-located users are the smallest group of users of BRICS-sourced DSI and the second smallest group of users of G77-sourced DSI.

Second, OECD DSI is used nearly 3 times more than either BRICS-sourced DSI or G77-sourced DSI. This is shown by the difference in the height of the bars. This is likely due mostly to the fact that there is simply a lot more OECD-sourced DSI in the databases than DSI from the other blocs. Many negotiators feel strongly that DSI sourced from mega-biodiverse countries is inherently more usable and valuable than DSI from other sources. The data shown here do not support this; otherwise G77 or BRICS DSI, even though fewer in the database, should be used more than OECD DSI.

Third, the graph also shows that there are important gaps between the different blocs. There are fewer total DSI-related publications coming from users (authors) located in G77 and BRICS compared to OECD-based users (∼30–40% fewer). The use of DSI and, likely, biological research in general has lower total output as compared to OECD countries. However, the scale of the data also shows that G77- and BRICS-based authors are still quite scientifically productive. Finally the asterisks indicate the use of G77 or BRICS-sourced DSI by OECD-located users. These users are the primary intended targets of the CBD discussions on DSI and benefit sharing even though these are the smallest blocks of use. It is critical that efforts to try to capture benefit sharing from these 2 small blocks do not disrupt the “self-use” of BRICS and G77-based authors, which could potentially inhibit the biological research community and, with it, opportunities for sustainable economic development in these countries. In other words, policymakers would be wise to ensure that a shot aimed at benefit sharing does not backfire on their own scientists.

The fourth question we were able to investigate with this dataset is the geographical interconnectedness between DSI researchers. To this end, network diagrams were built and which countries are using a given country's DSI (i.e., the countries to which country X is providing DSI. The “using” i.e., network displays the countries whose DSI are being used by country X's scientists. These data are also helpful to show that both neighbouring and distant countries use DSI from many countries.

**Figure 4.**
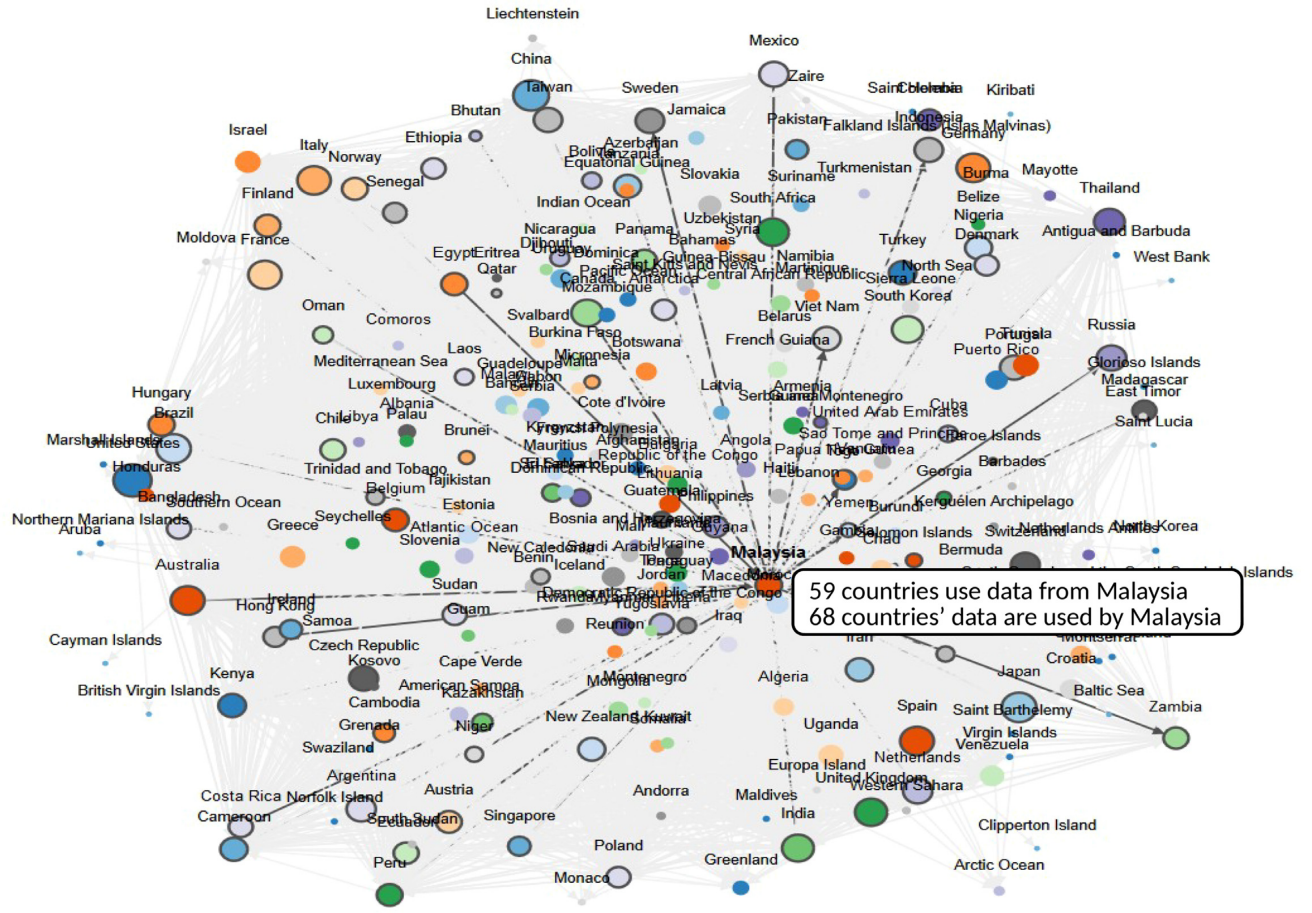
A network diagram displaying country-based DSI provision and use patterns. Data from Malaysia were selected as an example. 59 countries are using data from Malaysia; Malaysian scientists are using DSI from 68 countries. Neither the length of the connecting arrows nor the clustering reflects a statistical or quantitative relationship because the clustering algorithm is based on a random distribution. Each county's circle is proportional in size to the number of other coutries that use its DSI. An interactive chart is available on the web platform under Graph 6.3 [12].

In Fig. 4, DSI provisioning and use for Malaysia, which is both a G77 member and a mega-biodiverse country, is shown as an example. In Fig. 4, many LMICs (and not just HICs) use DSI from Malaysia, e.g., Zambia, India, Peru, and Mexico, scientists in Malaysia use DSI from 68 countries. Again, here there is no evidence of a provider-use relationship in DSI usage. Rather, Malaysian scientists use (cite in publications) DSI from a wide variety of countries and economic settings including Germany, Norway, Costa Rica, and Ghana. These data complement the data presented in Fig. 3, which suggests that, although scientists use their own national DSI more frequently, when they use foreign DSI they do not seem to be primarily using DSI from biodiversity-rich countries but rather DSI from across the world without any clear geographical or economic clustering patterns.

**Figure 5.**
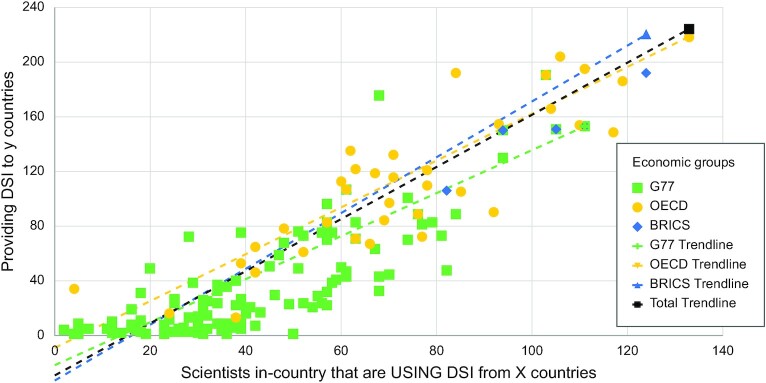
Relationship between use and provision of DSI for every country. The x-axis displays the use of DSI by a country's scientists and the y-axis displays the provisioning of DSI by a given country. An interactive chart is available on the web platform under Graph 6.4.

A final question that we addressed is what the overall providing-use relationship is for every country and whether there is a global trend to this relationship. Indeed, given the linear trend displayed in Fig. 5, it seems that many countries provide DSI to and use DSI from a roughly equal number of countries. In other words, if scientists from a given country are providing DSI, they are often using DSI at a similar level. However, small countries, especially LMICS, with accordingly smaller datasets and scientific communities, tend to cluster in the bottom left of the graph, meaning that they have little provisioning of DSI (i.e. providing access to GR that is subsequently sequenced) and even less scientific use of DSI.

## Discussion

Preconceptions in policymaking can be tested by looking at empirical data. Because there is a central repository for DSI, namely, the International Nucleotide Database Collaboration, global analyses can be conducted to inform the debate around benefit sharing from DSI and test hypotheses. The data presented here show that the concept of a user-provider dichotomy from provision by LMICs to use by HICs for DSI is rejected.

This suggests that if an ABS policy mechanism for DSI incorrectly assumes a unidirectional provider-user relationship in which benefits (non-monetary and monetary) flow from HICs to LMICs, this will only occur in some instances and, indeed, based on Fig. 3, is the least frequent type of DSI use. Given current political discussions, LMIC-sourced DSI would be the most likely to fall under an ABS regime, but it is LMIC researchers who are the predominant users of this DSI (Fig. 3). Thus, in a bilateral DSI ABS system (where benefit sharing is based on individual sequence records from a given country and benefit sharing is based only on an individual sequence), scientists working in LMICs will have benefit-sharing obligations for their use of other LMIC- and HIC-sourced DSI. This could have unintended consequences and harm scientists in LMICs because the DSI they use the most will require benefit sharing (Fig. 3, 2 left bars) while other DSI will likely not require benefit sharing.

Scientists in LMICs are often resource-limited and have more personnel and infrastructure constraints than scientists working in HICs. If DSI policymakers do not recognize and encourage the “self-use” of DSI, i.e., the use of that country's own DSI by in-country scientists, then they could potentially do great harm to scientists in LMICs, which could have long-term implications for their domestic bioeconomy strategies and broader research and innovation goals. There are indeed important inequalities across the globe, but a DSI ABS system should try to reduce these inequalities rather than exacerbate them.

The global goal, in our view, ultimately should be to increase the scientific output and generation of DSI from G77 and BRICS countries to levels similar to those observed within the OECD and shown in Fig. 3 (i.e., bring the 2 left bars up to levels similar to that of the right bar). Increased research capacity in LMICs would have global benefits, and global biodiversity knowledge gaps, including those identified by the Global Biodiversity Framework, could be better filled. To do this, any DSI policy mechanism should recognize the existing divide and encourage DSI use, publication, and collaboration, perhaps explicitly dedicating significant capacity-building to scientifically levelling the DSI playing field. This would be a much different approach than a “lock-it-up and control it” approach to DSI, which would negatively affect researchers everywhere on the globe and especially those in LMICs.

Furthermore, provisioning, use, and reuse of DSI as interpreted through DSI citation in publications is not a one-way street but rather a multi-directional traffic circle of data flowing in many different directions amongst all countries of the world. DSI is used by neighbouring countries and distant countries, by LMICs and HICs alike without any clear patterns or regional or economic trends (Fig. 4). Additionally, the network diagram reminds us that scientists working in developing countries, as well as the biological diversity of developed countries, are often overlooked in political discussions that oversimplify provision and use of biological data.

The sole clear trend observed in this analysis is that providing and using tend to go hand in hand (Figs 2 and 5). Large countries tend to use and provide a relatively large amount of DSI and smaller countries use and provide less, but trends based on development status (or, indirectly, the presence of mega-biodiversity) were not detected. In general, the relationship between use and provision seems to be relatively linear and not biased towards HICs but, instead, slightly biased towards LMICs (see G77 and BRICS trend lines, Fig. 5).

The dataset presented here is not a comprehensive dataset of all publications citing DSI but is limited to the open-access publications available for text mining in ePMC, as well as other dataset limitations explained in [11]. Furthermore, we note that the dataset would be further improved if the country of origin information (14.6% of sequences in this dataset had such information) provided by scientists submitting sequence data to INSDC were more consistent. Encouragingly, INSDC recently announced a new requirement for provenance information [14, 16]. With improved geographical and temporal information, clearer references to regional conditions would be possible and thus more valid scientific statements and analyses will be possible. For example, gene-function relationships could be mapped more precisely with climatic, geological, or atmospheric features.

However, acknowledging the aforementioned limitations, this analysis is the largest and only comparison of this size and perspective to date and represents a novel attempt to bring data and a new perspective on DSI to the policymaking process. We encourage other groups to expand and build upon this dataset for other policy environments and to reuse and complement these data with additional perspectives. Future studies are planned that will expand this dataset to include closed-access publications and the patent system [15], where greater insights on potential commercial use of DSI can be assessed. Furthermore, future assessments will expand the baseline datasets to include larger sequence datasets such as from the INSDC SRA, include a more expansive set of open-access publications, and provide first analyses on the field of study and taxonomic patterns.

## Potential Implications

Future political decisions around how to handle DSI should account for the complexity of geographical provision and use trends presented here. A DSI framework that requires benefit sharing from individual sequences should anticipate the high level of LMIC-LMIC DSI provision and use and high level of HIC-HIC DSI provision and use. Benefit-sharing expectations should be adjusted accordingly. If policymakers do not want to require LMICs to provide benefits to other LMICs, then a simple, decoupled multilateral mechanism should be considered that decouples access and use of individual sequences from benefit-sharing requirements and instead requires benefits further downstream in the value chain.

Policymakers also need to appreciate the tremendous contribution towards non-monetary benefit sharing that these global biological and publication databases make towards broader CBD goals and towards the UN Sustainable Development Goals. They should build in incentives in any DSI beneft-sharing framework to support these contributions. When policymakers meet in June 2022 to make a decision on DSI and the Global Biodiversity Framework, we hope these data will make a constructive contribution to evidence-based DSI policymaking.

Finally, these data also raise a fundamental question about the current ABS frameworks already in place for GR, especially the Nagoya Protocol. For GR there is no central repository for movement across national borders as there is for DSI, but these data suggest that the provider-user relationship, or the lack thereof, for GR could follow similar patterns to those observed for DSI. If so, this could suggest that the existing bilateral system and the predominance of user checks in HICs (rather than globally) is perhaps not the most appropriate way to ensure benefit sharing. While provocative, this could suggest that policymakers, in the future, ought to revisit ABS frameworks from the bottom up.

## Methods

The methods used in this article are described in the companion paper, which is a Data Note in the same issue of this journal [11].

## Data Availability

The dataset, figures, supplemental figures, and web application are available at http://wildsi.ipk-gatersleben.de. SQL queries to generate the figures are available in the *GigaScience* GigaDB repository [16].

## Abbreviations

ABS: access and benefit sharing; AN: accession number; BRICS: Brazil, Russia, India, China, South Africa; CBD: Convention on Biological Diversity; COP15: 15th Conference of the Parties to the Convention on Biological Diversity; DSI: digital sequence information; ePMC: European PubMed Central; G77: the Group of 77, representing mostly low-income countries; GR: genetic resources; HICs: high-income countries; INSDC: International Nucleotide Database Collaboration; LMICs: low- and middle-income countries; OECD: Organization of Economic Cooperation and Development; SRA: Sequence Read Archive; UN: United Nations.

## Competing Interests

The authors declare that they have no competing interests.

## Funding

This publication was made possible by the research project WiLDSI (Wissenschaftliche Lösungsansätze für Digitale Sequenzinformation) funded by the German Federal Ministry of Education and Research (BMBF) under funding code 031B0862.

## Authors' Contributions

A.H.S. wrote the manuscript. A.H.S., G.C., J.F., and M.L. designed the research with critical input from I.C. and P.O. M.L. and P.H. designed and programmed the data platform. All authors reviewed and edited the manuscript.

## Supplementary Material

giab085_GIGA-D-21-00228_Original_Submission

giab085_GIGA-D-21-00228_Revision_1

giab085_Response_to_Reviewer_Comments_Original_Submission

giab085_Reviewer_1_Report_Original_Submission Takeru Nakazato, Ph.D. -- 9/8/2021 Reviewed

giab085_Reviewer_2_Report_Original_Submission Michael Fire, Ph.D -- 9/10/2021 Reviewed
