# Supplementary material for: Myth-busting the provider-user relationship for digital sequence information
Source: Gigascience. 2021 Dec 29;10(12):giab085. doi: 10.1093/gigascience/giab085 (PMC8716360; doi:10.1093/gigascience/giab085)
Supplement: giab085_Response_to_Reviewer_Comments_Original_Submission [file giab085_response_to_reviewer_comments_original_submission.pdf]

Author's Response To Reviewer Comments

Close

Response to Reviewers

Dear Dr. Scott Edmunds and anonymous reviewers,

Thank you for the positive response to our manuscript, the helpful comments, and suggested improvements. We believe we have been able to address all of the reviewer's comments, which we respond to individually below. Most substantially, we have re-designed figure 1 and improved figure 2 per the suggestions of reviewer 2. Additionally, we have added several new sentences to address reviewer 1's concerns and conducted further typo checks and proofreading as recommended by reviewer 2.

We hope, with these edits, the manuscript will be appropriate for tandem publication with the "dataset paper" by Lange et al.

With our best regards,
Amber Hartman Scholz on behalf of the co-authors

Reviewer reports and responses:

Reviewer #1: The authors extract country names from gene sequence data in "traditional" ENA database and compare them with the country names of submitters to provide data-driven proof of the myth about the relationship between providers-users relationships for digital sequence information. I also implicitly believe in that myth. This verification has important implications for future handling of DSI. There is much room for debate in this manuscript, but I think it is important to ask the world as soon as possible. I make some points about this manuscript.

1. The Nagoya Protocol has prompted a major shift in the use of genetic resources. I think authors should also discuss the year of registration for digital sequence information. (Specifically, before and after the Nagoya Protocol) (It is desirable to provide actual data, but this time I will only seek the views of the author.)

At the present moment, there is no international consensus on whether digital sequence information (DSI) (at least that which is available from public databases) falls under an international legal framework for access and benefit-sharing. Therefore, there is no date that we can cite in the manuscript. However, as discussed in the introduction, a potential new access and benefit-sharing framework designed for DSI is actively being considered by Parties to the CBD. We have lightly edited the text to make this point 100% clear and added a new phrase "This decision is contested but widely expected to be resolved in some form.." and updated the dates for COP15 to April 2022.

2. The authors focus only on ENA's "traditional" gene sequence information (data corresponding to NCBI's GenBank). Regarding digital sequence information, NGS is currently being actively used, and data is also being accumulated in SRA (sequence read archive) at a tremendous pace. In addition, recent MinION devices can acquire digital sequence information on the spot without taking genetic resources out of the country. The lack of this data is a flaw in this manuscript, but at least the authors can discuss it in the manuscript, I think.

This paper builds off research done in Rohden et al. in which the "traditional" INSDC sequence dataset was analyzed. Thus, the same starting point for that study was used in this manuscript for consistency and comparison purposes which we hoped would be particularly useful for the policymakers that are already familiar with the Rohden et al. study which was requested by and produced for the Parties to the CBD as part of the inter-sessional process. Furthermore, in the interest of transparency, this project was

conducted on a shoestring budget as a pilot project to see whether we could make new contributions to the policy process.

However, the reviewer rightly points out that inclusion of the SRA dataset would provide more information and statistical power. However, the data in the “traditional” sequence dataset are y more likely to actually be the dataset that policymakers are interested in because the SRA dataset is more targeted towards re-sequencing of genomes and metagenomes. While this is rapidly evolving, our thinking was that the traditional dataset was most representative of biological diversity and thus the most relevant for the policy topic presented here.

For future work, we are considering incorporating the much larger SRA dataset. And, we are developing new methods that will allow for much larger publication datasets in a future analysis. As this will require table merges with billions of records, there are some technical limitations that are still being explored.

The sentence describing future work has been edited to address this point on p.11 “Furthermore, future assessments will expand the baseline datasets to include larger sequence dataset such as from the INSDC Short Read Archive, include a more expansive set of open access publications, and provide first analyses on the field of study and taxonomic patterns.”

3. In recent years, there has been a movement called "museomics" that extracts DNA from museum specimens and obtains digital sequence information. Museomics contributes to Ancient DNA and Taxonomic clarification. ENA/GenBank/DDBJ also has a "specimen_voucher" field, which already has hundreds of thousands of digital sequence information with this data (https://doi.org/10.3897/biss.5.73787). Does this have any effect on this study? Should DSI from museum specimen be excluded from the statistical processing in this study? (Authors don't have to mention this in the manuscript)

In Rohden et al., we noted that three types of metadata fields are available to connect sequence data to the original genetic resource: specimen_voucher, culture_collection, and bio_material. At present, only 4% of sequence entries use any of these three metadata fields, thus a relatively small fraction of the sequence dataset.

Furthermore, specimens that use these metadata fields might or might not fall out of the legal scope of the CBD’s requirements for benefit-sharing. It would be inappropriate to, as the reviewer implies, exclude all specimen_voucher-associated sequences as these genetic resources could just as easily be from ancient DNA or have been acquired post-1992 (effective date of the CBD) or post-2014 (effective date of the Nagoya Protocol). In other words, excluding these or other sequence data would neither have a significant statistical effect (less than 4% of the dataset) nor be legally appropriate given that sequences could come from Nagoya/CBD-relevant material.

Reviewer #2: The paper is interesting and easy to follow, and the interactive charts are useful and make it easy to interact with the data. Moreover, the implications and contributions of the study are clear. However, the paper needs to be carefully be edit and proofread in order to make it ready for publication.

Thank you for the helpful editing and proofreading suggestions here and below which we have implemented in the manuscript in track changes.

Minor Issues:
The paper needs to be carefully proofread and edited

Done.

The layout of Figure 1 isn't good. I recommend using subplots with subtitles. Remove of countries with very low percentage. In addition, the caption of this figure needs to be improved.

Figure 1 has been re-designed as a distribution diagram and now replaces the three pie charts. The caption has also been revised.

Figure 2. Beside the countries colored with pink. It is hard to distinguish between the values of different countries. Although this figure emphasis that the ratio is mostly even, I personally think, it would be better to use different color scheme, or normalize the values, so the difference among countries will more noticeable.

As the reviewer correctly points out, the graph is intended to emphasize that the ratio is mostly even. We have re-made the figure with a new color scheme to make the differences (although still minimal) more pronounced and hope this improves the visual interpretation. Indeed, this new color scheme particularly enables more coloring in the slightly darker blue (corn blue) countries such as Bolivia or southwestern African countries.

The paper's layout has unused free space in pages 5 and 6.

We believe this can be corrected during the subsequent editorial and layout process after re-submission and defer to the editorial staff.

The quality of figures throughout the paper need to be improve.

The figures were originally generated as .png files. We have significantly re-worked Figure 1 and 2 as requested by the reviewer and replaced all figures (except figure 4) with vector graphics which will be uploaded and submitted as separate files. Figure 4 is generated by "hovering over" the data for Malaysia and is thus the result of a user-experience interaction. Thus, unfortunately, for this figure, it is not possible to generate a vector graphic.

In the track-changes version of the re-submitted manuscript we have still included low-quality screenshots of the revised figures for convenience and ease of reviewing the manuscript. Please note these low-quality screenshots embedded in the MS Word document are intended to be removed in the copyediting process.

Typos (some examples):

All typos have been corrected. Many thanks to reviewer 2 for the keen eyes! Unless otherwise noted, they have been addressed in track changes.

- Abstract: CBD) -> CBD,
- P. 2: "for user checks [4]. (Countires"
- P. 3: originated. (Note

This seems, in our opinion to be grammatically correct as-is.

- P. 3: "use" -> "use."
- Figure 2: graph 3.4 -> Graph 3.4 (why call this Graph?)

Graph 3.4 refers to the numbering of the graphs/figures available through the online data platform, which differ from the figure numbering in the manuscript. In fact, every figure in the manuscript has a similar sentence at the figure legend to refer the reader to the online data platform and indicate the numbering on that platform.

@Editorial staff: Would it be possible to embed a hyperlink to the data platform in the figure legend? We have added hyperlinks in the text and hope they will function still for the online manuscript.

- P. 8 "DSI (/country "

/country is not a typo. This "/" is indicative that this is a formal metadata field associated with sequence entries. We have attempted to show this more clearly in the caption. This is fully explained earlier in the manuscript on p.3.

- P. 8: figure 3 -> Figure 3
- Figure captions are with different formats.
- Figure 4 caption is in a mislocated
- P. 12: Figure 2 and 5 -> Figures 2 and 5

Close
