# Supplementary material for: Myth-busting the provider-user relationship for digital sequence information
Source: Gigascience. 2021 Dec 29;10(12):giab085. doi: 10.1093/gigascience/giab085 (PMC8716360; doi:10.1093/gigascience/giab085)
Supplement: giab085_Reviewer_1_Report_Original_Submission — Takeru Nakazato, Ph.D. -- 9/8/2021 Reviewed [file giab085_reviewer_1_report_original_submission.pdf]

Reviewer Report

Title: Myth-busting the provider-user relationship for digital sequence information

Version: Original Submission **Date:** 9/8/2021

Reviewer name: Takeru Nakazato, Ph.D.

Reviewer Comments to Author:

The authors extract country names from gene sequence data in "traditional" ENA database and compare them with the country names of submitters to provide data-driven proof of the myth about the relationship between providers-users relationships for digital sequence information. I also implicitly believe in that myth. This verification has important implications for future handling of DSI. There is much room for debate in this manuscript, but I think it is important to ask the world as soon as possible. I make some points about this manuscript.

1. The Nagoya Protocol has prompted a major shift in the use of genetic resources. I think authors should also discuss the year of registration for digital sequence information. (Specifically, before and after the Nagoya Protocol) (It is desirable to provide actual data, but this time I will only seek the views of the author.)
2. The authors focus only on ENA's "traditional" gene sequence information (data corresponding to NCBI's GenBank). Regarding digital sequence information, NGS is currently being actively used, and data is also being accumulated in SRA (sequence read archive) at a tremendous pace. In addition, recent MinION devices can acquire digital sequence information on the spot without taking genetic resources out of the country. The lack of this data is a flaw in this manuscript, but at least the authors can discuss it in the manuscript, I think.
3. In recent years, there has been a movement called "museomics" that extracts DNA from museum specimens and obtains digital sequence information. Museomics contributes to Ancient DNA and Taxonomic clarification. ENA/GenBank/DDBJ also has a "specimen_voucher" field, which already has hundreds of thousands of digital sequence information with this data (https://doi.org/10.3897/biss.5.73787). Does this have any effect on this study? Should DSI from museum specimen be excluded from the statistical processing in this study? (Authors don't have to mention this in the manuscript)

Methods

Are the methods appropriate to the aims of the study, are they well described, and are necessary controls included? Choose an item.

Conclusions

Are the conclusions adequately supported by the data shown? Choose an item.

Reporting Standards

Does the manuscript adhere to the journal's guidelines on minimum standards of reporting? Choose an item.

Choose an item.

Statistics

Are you able to assess all statistics in the manuscript, including the appropriateness of statistical tests used? Choose an item.

Quality of Written English

Please indicate the quality of language in the manuscript: Choose an item.

Declaration of Competing Interests

Please complete a declaration of competing interests, considering the following questions:

- Have you in the past five years received reimbursements, fees, funding, or salary from an organisation that may in any way gain or lose financially from the publication of this manuscript, either now or in the future?
- Do you hold any stocks or shares in an organisation that may in any way gain or lose financially from the publication of this manuscript, either now or in the future?
- Do you hold or are you currently applying for any patents relating to the content of the manuscript?
- Have you received reimbursements, fees, funding, or salary from an organization that holds or has applied for patents relating to the content of the manuscript?
- Do you have any other financial competing interests?
- Do you have any non-financial competing interests in relation to this paper?

If you can answer no to all of the above, write 'I declare that I have no competing interests' below. If your reply is yes to any, please give details below.

I declare that I have no competing interests

I agree to the open peer review policy of the journal. I understand that my name will be included on my report to the authors and, if the manuscript is accepted for publication, my named report including any attachments I upload will be posted on the website along with the authors' responses. I agree for my report to be made available under an Open Access Creative Commons CC-BY license (http://creativecommons.org/licenses/by/4.0/). I understand that any comments which I do not wish to be included in my named report can be included as confidential comments to the editors, which will not be published.

Choose an item.

To further support our reviewers, we have joined with Publons, where you can gain additional credit to further highlight your hard work (see: https://publons.com/journal/530/gigascience). On publication of this paper, your review will be automatically added to Publons, you can then choose whether or not to claim your Publons credit. I understand this statement.

Yes Choose an item.
